# Combining Transarterial Embolization and Percutaneous Cryoablation for Early-Stage Renal Cell Carcinoma: Embolization Materials and Impacts of Tumor Size

**DOI:** 10.3390/tomography10110130

**Published:** 2024-11-07

**Authors:** Miki Terauchi, Tsuneo Yamashiro, Shungo Sawamura, Shingo Koyama, Noboru Nakaigawa, Keiichi Kondo, Hisashi Hasumi, Kazuhide Makiyama, Daisuke Utsunomiya

**Affiliations:** 1 Department of Interventional Radiology, Japan Organization of Occupational Health and Safety Yokohama Rosai Hospital, 3211 Kozukuechou, Kohoku-ku, Yokohama 222-0036, Japan; 2 Department of Diagnostic Radiology, Yokohama City University Graduate School of Medicine, 3-9 Fukuura, Kanazawa-ku, Yokohama 236-0004, Japan; clatsune@yahoo.co.jp (T.Y.); sawa0808@yokohama-cu.ac.jp (S.S.); singo109@yokohama-cu.ac.jp (S.K.); d_utsuno@yokohama-cu.ac.jp (D.U.); 3 Department of Urology, Yokohama City University Graduate School of Medicine, 3-9 Fukuura, Kanazawa-ku, Yokohama 236-0004, Japan; nakaigan@yokohama-cu.ac.jp (N.N.); kkurouro@yokohama-cu.ac.jp (K.K.); hasumi@yokohama-cu.ac.jp (H.H.); makiya@yokohama-cu.ac.jp (K.M.); 4 Department of Urology, Kanagawa Cancer Center, 2-3-2 Nakao, Asahi-ku, Yokohama 241-8515, Japan; 5 Kanazawa-Hakkei Kondo Urology Clinic, 19-14 Seto, Kanazawa-ku, Yokohama 236-0027, Japan

**Keywords:** renal cell carcinoma, computed tomography, percutaneous cryoablation, transarterial embolization

## Abstract

Background/Objectives: Our aim was to compare the complication rates of different embolization materials (absolute ethanol and gelatin sponges) used for combined transarterial embolization (TAE) and to investigate the impact of tumor size on operative time and cryoneedle use during percutaneous cryoablation (PCA). Methods: We treated 27 patients (9 women and 18 men; mean age, 74 years) with 28 early-stage (T1a) renal cell carcinoma (RCC) lesions using combined TAE and PCA between September 2018 and January 2021. During TAE, 15 lesions in 14 patients were embolized using mixed absolute ethanol and iodized oil. The remaining 13 lesions (in 13 patients) were embolized using a gelatin sponge followed by iodized oil. The PCA was performed within 3 to 21 days of the TAE. We compared complications between the TAE subgroups (i.e., absolute ethanol and gelatin sponge) and assessed potential correlations between tumor size and the operative time of the PCA. Results: All patients were successfully treated by combined TAE-PCA. Local control was achieved for all patients (monitoring period, 1–48 months; median, 28 months). Although the effect of TAE did not differ between subgroups, a significantly higher number of patients in the absolute ethanol group experienced intraprocedural pain than in the gelatin sponge group (*p* < 0.05). The operative time of the PCA was significantly correlated with the size of the RCC lesion (*p* < 0.01). The number of cryoneedles used for the PCA was also correlated with the size of the RCC lesion (*p* < 0.0001). Conclusions: For TAE prior to PCA for early-stage RCC, gelatin sponges can replace absolute ethanol to reduce intraprocedural pain. Tumor size correlates with operative time and the number of cryoneedles needed for PCA, which suggests the total medical cost for PCA therefore varies based on the tumor’s size.

## 1. Introduction

With continuing developments in imaging technology, the number of incidental renal cell carcinomas (RCCs) detected has increased, most of which are early-stage RCC [1]. Partial nephrectomy is typically recommended for early-stage RCC, with radical nephrectomy being an option for situations in which partial nephrectomy is technically difficult [2,3,4]. However, due to high perioperative risk, deteriorated renal function, or the presence of multiple tumors, some patients with early-stage RCC cannot undergo partial or radical nephrectomy.

The number of patients with early-stage RCC who are treated with thermal ablation has increased recently [5]. Compared with partial nephrectomy, thermal ablation has a lower complication rate, shorter hospital stay, and allows for preserved renal function [6,7,8,9,10,11,12,13]. The European Society for Medical Oncology has stated that thermal ablation techniques for early-stage RCC, including cryoablation (CA) and radiofrequency ablation (RFA), are useful therapeutic options for patients who are considered high risk by surgical management and those with low renal function, multiple RCC lesions from hereditary disease, or absence their contralateral kidney [14]. Other guidelines describe thermal ablation as an alternative to conventional surgery for patients with early-stage RCC [3,4]. The American Urological Association (AUA) recommends a percutaneous approach to thermal ablation over a laparoscopic approach because of the shorter operative time, more rapid postoperative recovery time, and because general anesthesia is not required [2]. Since no randomized controlled trials have compared CA with RFA, the best method for performing thermal ablation remains unclear [2].

Percutaneous CA (PCA) is considered to be as safe as laparoscopic CA, with equivalent oncologic efficacy and a shorter hospital stay [15]. There are also some reports stating that PCA is superior to partial nephrectomy from a medical cost standpoint [5,16,17,18,19]. Although a PCA is a shorter procedure than a partial nephrectomy [9,10,11,12,13], it is unclear which clinical factors influence the operative time for PCA. It seems logical that the operative time would be affected by the size of the target lesion and that more PCA cryoneedles would be needed for larger RCC lesions.

Percutaneous selective transarterial embolization (TAE) is often performed before PCA for early-stage RCC, particularly in Japan, in order to enhance the visualization of the target lesion, decrease blood flow and prevent hemorrhage, and decrease the cold-sink effect of PCA. There are multiple reports describing the efficacy and safety of TAE preceding PCA for RCC [20,21,22,23,24,25,26,27,28,29]. The embolic material used for TAE varies and may include microparticles, coils, iodized oil, absolute ethanol, gelatin sponges, and combinations of these materials. Microparticles, coils, and absolute ethanol are considered to be permanent embolic materials, whereas a gelatin sponge is temporary. Absolute ethanol has been widely used for TAE in several renal diseases, including RCC, renal angiomyolipoma, renal graft intolerance syndrome, polycystic kidney disease, and vascular malformations [30]. Gelatin sponges have also frequently been used to achieve hemostasis and preoperative devascularization in various types of renal tumors [30]. There are multiple reports describing the efficacy, safety, and complications of absolute ethanol and gelatin sponges for TAE prior to PCA for RCC [24,25,26,27,28]. Since absolute ethanol causes painful endothelial damage during TAE [30,31], the temporary RCC embolization provided by a gelatin sponge may be a better option from the standpoint of intraprocedural pain.

In this study, we aim to compare two embolic materials used for TAE—absolute ethanol and gelatin sponges—in terms of their efficacy and intraprocedural pain, and to investigate the correlation between the size of the targeted RCC and PCA operative time and the number of cryoneedles used during PCA.

## 2. Materials and Methods

### 2.1. Patients

This retrospective, observational study was approved by the Institutional Review Board of Yokohama City University (B210500035). Written informed consent was waived for this study.

This was a retrospective study analyzing a single group of patients; therefore, specific guidelines such as those defined by the Enhancing the QUAlity and Transparency Of health Research (EQUATOR) Network did not apply. We obtained the records of 28 patients who underwent combined percutaneous selective TAE and PCA for early-stage RCC (T1a; ≤4 cm in diameter) at our institution between September 2018 and January 2021. One patient was excluded from the analysis since neither absolute ethanol nor gelatin sponges were used for their TAE. The 27 patients were referred by urologists to the radiology department of our institution when they were deemed unsuitable for surgery under general anesthesia because of comorbidities (respiratory failure, n = 4; advanced age [≥85 years], n = 3; cardiac failure, n = 2; malnutrition, n = 1; advanced breast cancer, n = 1; obesity, n = 1; renal dysfunction, n = 6), the location of the RCC lesion (not amenable to partial nephrectomy, n = 1), a previous removal of the contralateral kidney (n = 4), multiple RCC lesions due to a hereditary disease (von Hippel-Lindau syndrome [VHL], n = 2; Birt-Hogg-Dubé syndrome [BHD], n = 1), or at the patient’s request (n = 1).

Preoperative staging of the target lesion was performed using computed tomography (CT) in all patients. The diagnosis of RCC was either pathologically confirmed by a percutaneous biopsy performed prior to TAE or clinically suspected based on family history, imaging findings, or a prior surgery for RCC.

### 2.2. TAE Technique

All TAE procedures were performed by radiologists board-certified by the Japan Radiological Society (M.T., S.S., S.K.). Catheterization for the TAE was performed using the right common femoral artery in all patients. The puncture points were draped in standard sterile fashion. Local anesthesia was administered using 1% lidocaine (Sandz Pharma Inc., Tokyo, Japan). For all patients, a 4- or 5-F arterial sheath (Terumo Clinical Supply Company, Ltd., Gifu, Japan) was inserted under fluoroscopic guidance using an 18 G needle and a 0.035-inch wire. All procedures were performed using a fluoroscopic machine (Artis zee BA Twin; Siemens Healthineers Inc., Erlangen, Germany).

After a 4- or 5-F diagnostic catheter (Shepherd Hook; MEDIKIT Inc., Tokyo, Japan) was inserted into the renal artery, angiography was performed to visualize the target RCC lesion and to identify the feeding arteries using iohexol contrast (Hikari Pharmaceutical Co., Ltd., Tokyo, Japan). The feeding arteries for the target RCC were selected using a combination of a 1.9- to 2.2-F microcatheter (Sniper2, Progreatβ, Progreatλ, Terumo Clinical Supply Company, Ltd., Gifu, Japan; LOGOS GrandMaster; PIOLAX Medical Devices Inc., Yokohama, Japan) and a microwire. Once the end (tip) of the microcatheter was placed into the feeding artery, embolization under fluoroscopic guidance was performed using absolute ethanol (Pfizer Inc., New York, NY, USA) or a gelatin sponge (Spongel; LTL Pharma Inc., Tokyo, Japan). Iodized oil (Lipiodol; Guerbet Japan Inc., Tokyo, Japan) was mixed and stirred with the absolute ethanol or injected before embolization using the gelatin sponge. In June 2020, we changed our standard embolization technique from the use of absolute ethanol to the use of gelatin sponges in order to avoid causing injection pain during TAE.

For all patients, TAE was deemed complete when the targeted RCC lesion “disappeared” on the angiography. Tumor enhancement from the iodized oil was confirmed using postoperative CT, with a combined fluoroscopy–CT scanner (SOMATOM Emotion 16; Siemens Healthineers), performed immediately after the TAE procedure.

### 2.3. PCA Technique

All PCA procedures were performed by radiologists board-certified by the Japan Radiological Society (M.T., S.S., S.K.). All patients underwent PCA under local anesthesia using 1% lidocaine (Sandz Pharma Inc., Tokyo, Japan). Some patients were sedated as needed using a combination of pentazocine (Sosegon; Maruishi Pharma Inc., Osaka, Japan) and hydroxyzine hydrochloride (Atarax-P; Pfizer Inc., New York, NY, USA). All procedures were performed using appropriate cleanliness techniques. Hydrodissection with saline combined with 2% iopamidol contrast (Hikari Pharmaceutical Co., Ltd., Tokyo, Japan) was performed when the tumor was close to other organs such as the intestinal tract, liver, spleen, pleura, or psoas muscle. If the tumor was located close to the renal pelvis or ureter, a ureteral catheter was placed in advance of the PCA procedure. Procedures were performed using a commercially available ablation machine (CryoHit; Galil Medical USA Inc., Arden Hills, MN, USA). Two types of cryoneedles were used, depending on the tumor diameter and shape (IceSeed or IceRod; Galil Medical USA Inc., Arden Hills, MN, USA).

The cryoneedles were inserted into the target RCC under CT fluoroscopy. The target RCCs were frozen using argon gas and thawed using helium gas. The freezing process created ice balls, which were recognized on the CT as spherical hypodense areas. The location and angles of the cryoneedles were carefully checked using CT fluoroscopy at puncture and at ablation.

For all RCC lesions, the PCA series consisted of 1st and 2nd ablations (15 min each), which were continuously performed with an interval of 5 min of passive thawing. Active thawing using helium gas was performed after the 2nd ablation procedure of the series.

The completion of PCA was confirmed upon viewing ice balls covering the target RCC lesions with margins of at least 6 mm.

### 2.4. Imaging and Laboratory Examination

The effect of TAE (the accumulation of iodized oil in the target RCC on post-TAE CT) was reviewed by 2 radiologists board-certified by the Japan Radiological Society (M.T. and T.Y.); both radiologists reached a consensus using a 3-point scale (1 = poor, 2 = fair, 3 = good). The CT characteristics of the iodized oil accumulation were rated as either 1 (poor), very limited enhancement in the RCC lesions or at the margins of RCC lesions; 2 (fair), heterogeneous or partial enhancement in the RCC lesions; or 3 (good), homogeneous and complete enhancement in the RCC lesions. The hypervascularity or hypovascularity of the target RCC was assessed by one of the interventional radiologists (M.T.) by viewing the angiography obtained at the TAE. The operative time and the occurrence of patient pain during the TAE were collected from the nursing record.

For PCA, the types and numbers of cryoneedles used, operative time, and performance of hydrodissection were collected from the PCA procedure record.

Blood was collected to assess pre-TAE and post-PCA hemoglobin (Hb) and the estimated glomerular filtration rate (eGFR) and C-reactive protein (CRP) level (determining hemorrhages, renal function, and inflammatory changes, respectively).

All patients were carefully monitored by urologists at an outpatient clinic at our institution after discharge.

### 2.5. Statistical Analysis

Continuous variables were expressed as a mean with standard deviation (SD). For comparing the 2 patient groups, the Mann–Whitney U test was used for continuous variables and the chi-square test for categorial variables. Correlations between 2 continuous variables were estimated using Spearman’s rank correlation coefficient.

For TAE, the between-group differences in clinical characteristics (e.g., operative time, reported pain, iodized oil accumulation) were statistically compared. For PCA, the influence of clinical characteristics (i.e., tumor location, necessity of hydrodissection, iodized oil accumulation, vascularity) on the operative time was evaluated. The correlations between tumor size and operative time were assessed for both TAE and PCA. The correlation between tumor size and the number of cryoneedles required was also assessed for PCA.

Changes in Hb, eGFR, and CRP (pre-TAE and post-PCA) were also examined.

A *p* value of 0.05 was considered statistically significant. All analyses were performed using JMP software, version 10.0.2 (SAS Institute, Cary, NC, USA).

## 3. Results

### 3.1. Patients

The patient characteristics are presented in Table 1. A total of 27 patients underwent TAE followed by PCA during the study period. In 17 patients, RCC was pathologically confirmed by a percutaneous biopsy performed prior to the TAE. The remaining 10 patients were clinically diagnosed: 5 patients had either hereditary RCC strongly presumed by a family history of VHL or BHD, 2 had an RCC suggested by a typical contrast-enhancement pattern on dynamic-enhanced CT with an increase in lesion size, and 3 had a local recurrence suspected after a prior partial nephrectomy with a pathologic diagnosis of RCC. One patient had two RCC lesions in a single kidney and was treated by two PCA procedures following a single TAE. Thus, the total number of RCC lesions treated and analyzed in this study was 28.

The mean (SD) patient age was 74 (16) years old (range, 32–93 years old). All TAEs and PCAs were successfully completed for the 28 RCC lesions in 27 patients. The mean (SD) tumor size was 24.8 (7.2) mm (range, 13–38 mm).

Within the observation period, no local recurrence was observed (mean [SD], 28 [14] months; range, 1–48 months). Three patients developed pulmonary metastases: two within 12 months and one within 1 month. Two patients died due to other reasons: one died of breast cancer at 8 months and the other of microscopic polyangiitis at 28 months.

### 3.2. Transarterial Embolization

**Figure 1:**
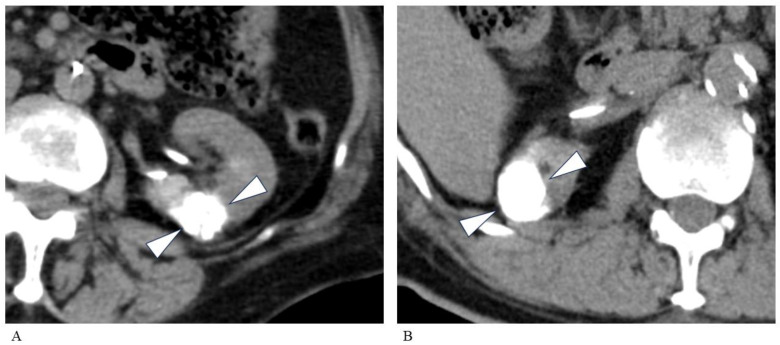
Comparison of different embolic materials. (**A**) A CT image of a 78-year-old woman with a left RCC. This image was taken immediately after TAE (mixture of absolute ethanol and iodized oil). A 19 mm RCC is clearly seen (arrowheads). The accumulation score was 3 (good). (**B**) A CT image of a 79-year-old man with a right RCC. A 23 mm RCC is clearly seen (arrowheads) after TAE (injection of iodized oil followed by gelatin sponge). There is no apparent difference in iodized oil accumulation compared with the TAE using absolute ethanol. The accumulation score was 3 (good). CT, computed tomography; RCC, renal cell carcinoma; TAE, transarterial embolization.

A total of 14 patients (15 RCC lesions) were treated with absolute ethanol TAE, while 13 patients (13 RCC lesions) were treated with gelatin sponge TAE (Figure 1). There was no significant correlation between tumor size and the time taken to complete the TAE. The frequency of pain was significantly higher in the absolute ethanol group than in the gelatin sponge group (*p* < 0.05). In the group that underwent embolization with absolute ethanol, no complications specific to the agent (such as an acute bronchospasm, disseminated intravascular coagulation, fatal cardiovascular collapse, or pulmonary hypertension) were identified except for pain. Neither hypervascularity vs. hypovascularity nor the choice of embolization material affected the time taken to complete the TAE.

There was a significant difference in lipiodol accumulation in tumors after the TAE, with absolute ethanol showing poor accumulation. When a multivariate analysis was added to the study, it became clear that differences in vascularity had a significant effect on lipiodol accumulation after TAE (three patients in the absolute ethanol group and one patient in the gelatin sponge group had hypovascular tumors).

The data associated with TAE are presented in Table 2.

### 3.3. Percutaneous Cryoablation

Hydrodissection was used for 22 of the 28 tumors but did not affect the time needed to complete the PCA.

Some patients experienced minor complications. Hematuria was observed after 14 procedures, one patient had frostbite at the puncture site, two had pneumothorax, one had a splenic hemorrhage, one had liver injury, and five had a small nearby hematoma; only the patient with the splenic hemorrhage required additional treatment (blood transfusion).

**Figure 2:**
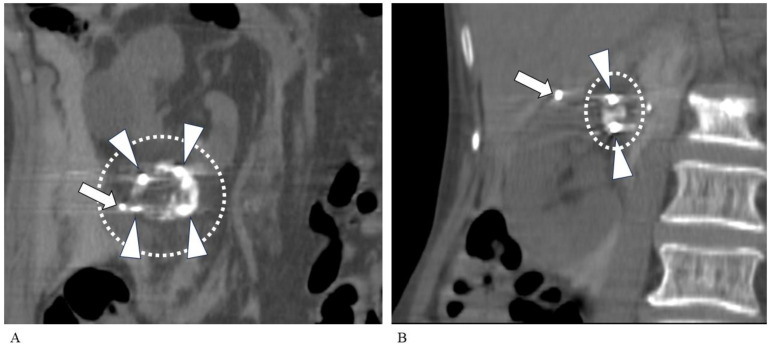
Differences in operative time and number of needles needed for tumors of different sizes. Coronal CT images taken 15 min after the second ablation. (**A**) An 86-year-old man with a left RCC visualized by iodized oil. The lesion is completely covered by a large ice ball (dashed oval). The tumor diameter was 34 mm, requiring 4 cryoneedles (arrowheads). The total operative time was 230 min. Arrow: needle for hydrodissection. (**B**) An 86-year-old woman with right-sided RCC. The tumor diameter was 13 mm, requiring 2 cryoneedles (arrowheads). A small ice ball is seen (dashed oval). The total operative time was 125 min. Arrow: needle for hydrodissection. CT, computed tomography; RCC, renal cell carcinoma.

**Figure 3:**
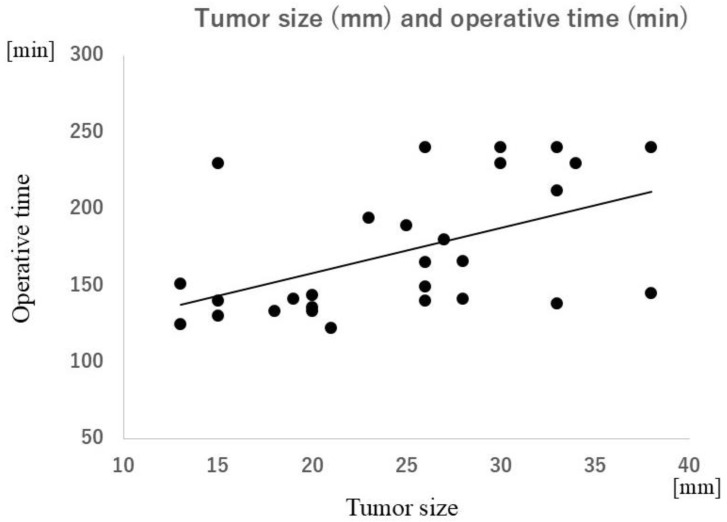
Correlation between tumor size and total PCA operative time. A significant positive correlation is observed (ρ = 0.55; *p* < 0.01). PCA, percutaneous cryoablation.

**Figure 4:**
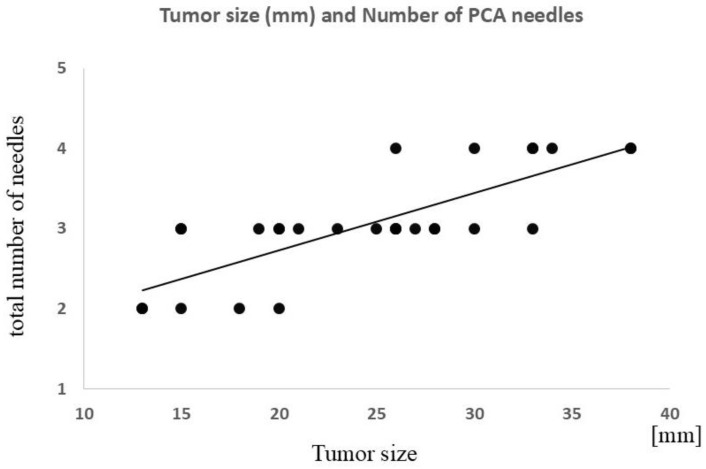
Correlation between tumor size and total number of PCA needles used. A significant positive correlation is observed (ρ = 0.78; *p* < 0.0001). PCA, percutaneous cryoablation.

There was no significant difference in operative time based on tumor location (i.e., top, middle, or bottom of the kidney). There was a significant correlation between tumor size and operative time (ρ = 0.55; *p* < 0.01) and between tumor size and the number of cryoneedles required (ρ = 0.78; *p* < 0.0001) (Figure 2, Figure 3 and Figure 4). The strength of the accumulation of lipiodol did not affect the time taken to complete the PCA, nor did hypervascularity vs. hypovascularity. Tumors size-needled using IceSeeds (mean, 18.2; SD, 2.9 mm) were significantly smaller than those needled using IceRods (mean, 26.2; SD, 7.3 mm) (*p* < 0.05). The PCA time was not affected by whether the embolization material was absolute ethanol or gelatin sponges.

There was no correlation between the operative time for PCA and the operative time for TAE.

All correlation measurements are presented in Table 3, except for the correlations between tumor size and operative time or the number of cryoneedles used, which are presented in Table 4.

### 3.4. Laboratory Analysis

In the entire patient cohort, post-PCA Hb significantly decreased by a mean value of 0.6 (SD of 0.8) g/dL (*p* < 0.001) compared to pre-TAE Hb levels. Post-PCA eGFR showed a tendency to decrease by 2.6 (SD 8.1) mL/min (*p* = 0.05) compared to pre-TAE eGFR. Post-PCA CRP significantly increased by 2.46 (SD 2.65) mg/dL (*p* < 0.0001) compared to pre-TAE CRP.

In the gelatin sponge group, post-PCA Hb significantly decreased by 0.4 (SD 0.7) g/dL (*p* < 0.05), and post-PCA eGFR also significantly decreased by 3.5 (SD 1.6) mL/min (*p* < 0.05). Post-PCA CRP significantly increased by 1.40 (SD 0.15) mg/dL (*p* < 0.001). One patient was excluded from post-PCA blood sampling because of a femoral neck fracture that occurred after the PCA. In the absolute ethanol group, post-PCA Hb significantly decreased by 0.8 (SD 0.3) g/dL (*p* < 0.01), post-PCA eGFR decreased by 2.1 (SD 2.8) mL/min (not significant), and post-PCA CRP significantly increased by 3.57 (SD 0.97) mg/dL (*p* < 0.001). The decreases in eGFR and Hb and increases in CRP were not significantly different between the absolute ethanol group and the gelatin sponge group.

The laboratory data are presented in Table 5.

## 4. Discussion

This study summarizes the data on patients with small RCC lesions (T1a) who underwent combined therapy with TAE and PCA at our institution. During TAE, the use of absolute ethanol evoked pain; this was not observed when gelatin sponges were used. For the PCA, tumor size (maximum diameter) was significantly correlated with operative time and the number of cryoneedles required. Comparing pre-TAE and post-PCA laboratory data showed that there was a significant increase in CRP and a significant decrease in Hb, but the different materials used for the TAE did not affect these changes. Based on these findings, we believe that a gelatin sponge should be used for TAE in place of absolute ethanol, and that tumor size is an important clinical factor for successful PCA treatment in patients with T1a RCC.

Currently, the embolic materials used for TAE prior to PCA vary among operators and institutions, and a few institutions perform PCA without a preceding TAE. Multiple previous reports have shown the positive effects of a TAE prior to PCA for RCC [20,24,25,26,27,28,29], mainly in terms of the visualization of tumor margins, decreased bleeding during the PCA, and a decreased cold-sink effect of tumor vessels.

At our institution, TAE is performed in all patients prior to PCA. Absolute ethanol, with iodized oil or iodized oil, followed by a gelatin sponge is used for our TAE procedures. We strongly believe that using the TAE procedure may have led to the safe PCAs without major bleeding at our institution. We could not find any research articles comparing gelatin sponges to absolute ethanol as embolic materials at a TAE prior to PCA for RCC. However, it is widely known that the arterial injection of absolute ethanol evokes pain [30]; this was confirmed in our study. Considering that no clinical parameters except for the presence of pain were significantly different between the two patient groups, and because the effect of the TAE could be temporary for the following PCA, gelatin sponges should be used at a TAE prior to planned PCA.

The AUA guideline recommends thermal ablation as an alternative to partial nephrectomy for small RCCs (T1a, particularly < 3 cm) [2]. Thermal ablation is superior to partial nephrectomy from multiple viewpoints: it has a low intraoperative conversion to radical nephrectomy, low perioperative transfusion rate, short hospital stay, and a similar metastasis-free survival rate. Percutaneous thermal ablation (e.g., PCA) is strongly recommended over laparoscopic thermal ablation due to its shorter operative time, more rapid recovery time, and because general anesthesia is not required, which results in high cost-effectiveness. In this study, good local control was achieved in all patients (there were no local recurrences), although the observation period was not long enough for some patients. One patient required a blood transfusion for a splenic injury incurred during their PCA; however, no other major complications occurred during PCAs. Local anesthesia was used for all patients undergoing PCA in this study. We therefore believe that the results of the PCA treatment are satisfactory, as stated by the AUA guideline [2].

Generally, the operative time for PCA is shorter than that of laparoscopic CA or partial nephrectomy [2,9,10,11,12,13]. One article reported no difference in operative time between PCA and laparoscopic CA; however, this was probably because both PCA and laparoscopic PCA were performed under general anesthesia [15]. In our study, targeting T1a RCCs only, there was a significant correlation between tumor size and operative time. The general condition of the treated patients was not good; therefore, PCA under local anesthesia with a short operative time was ideal for this population.

Based on our results, it is easy to surmise that the operative time will increase for larger tumors (such as T1b). In recent years, many reports and reviews of the treatment options for T1b RCCs have been published [26,32,33,34,35,36,37]. The National Comprehensive Cancer Network has described thermal ablation as an option for patients with T1b RCC for whom a partial or radical nephrectomy is not a suitable option [3]. PCA for patients with T1b RCC has a lower local control rate and higher complication rate than when it is used for T1a RCC. Also, PCA for patients with T1b RCC reportedly results in a poorer long-term prognosis than partial nephrectomy [4]. Based on our results, it can be predicted that larger RCC lesions will require longer operative times during their PCA, which cannot be performed with local anesthesia and therefore requires general anesthesia. In such situations, the advantages of PCA over partial nephrectomy or laparoscopic CA disappear, and even PCA would result in a long hospital stay and incur high medical costs for RCC treatment. It should also be noted that, for RCC in a solitary kidney, the choice to perform thermal ablation (including PCA) must be made carefully based on various clinical factors; a partial nephrectomy is still considered the standard treatment approach [38].

One possible approach to shortening the operative time of PCA for RCC would be to reduce the size of the RCC lesion to the T1a level, either by adjuvant chemotherapy or by using TAE techniques intended to decrease the size of the RCC lesion in advance of PCA. There is a report describing an aggressive reduction in tumor size with TAE using polyvinyl alcohol particles prior to PCA [22]. Considering the correlation between tumor size and operative time in this study, which targeted T1a RCCs only, a future therapeutic option for RCC may be a reduction in RCC size using TAE or chemotherapy prior to PCA.

This study has shown that more cryoneedles are needed at PCA with increasing tumor size. The cost of the PCA treatment varies based on the length of hospital stay and the type of anesthesia used (local or general). However, the most expensive medical device or drug used for combined TAE-PCA for RCC is the cryoneedle. Under the current Japanese public healthcare system, the medical fee for a PCA for RCC is paid for as a comprehensive payment. This means that if more cryoneedles are used at PCA, the cost of the PCA may exceed the fee paid to the hospital. In this study, we found that relatively large (>25 mm in diameter) RCCs often needed four needles. Currently, in Japan, a maximum of three cryoneedles are covered by the medical fee paid to hospitals; therefore, using four or more cryoneedles leads to extra (unpaid) costs for hospitals performing PCAs for RCC. If a PCA is superior to partial nephrectomy from the standpoint of medical costs, then adequate standard medical reimbursement should be continuously discussed to accelerate the use of PCAs for small RCCs, particularly in Japan. The cost of the cryoneedles may be further reduced by a fundamental, technical innovation of the needles themselves. For example, if a novel cryoneedle forms a larger ice ball with a single punctuation, the new needle would not only reduce the number of needles used at PCA, but also shorten the operative time, which may greatly contribute to minimizing the entire medical cost of a PCA for RCC.

There are several limitations to this study. First, the number of patients was limited. Second, this was a retrospective study, and the observation period after the PCA varied. Although the aim of this study was not to monitor the long-term efficacy of PCA, unpredicted events may have occurred after the short follow-up period at our institution. Third, pathologic results were not available for all patients, resulting in both general RCC and hereditary RCC being represented in our patient sample. Fourth, the severity of pain at the TAE was not evaluated using a standardized tool such as the Visual Analogue Scale; only the presence or absence of pain was documented during the injection of embolic materials. Fifth, there is a potential for selection bias in our study design, since the indication for combined TAE-PCA was based on clinician judgment and there was no control group. Finally, some patients did not undergo a biopsy for pathologic confirmation of an RCC, particularly those in whom RCC was considered likely because of heredity disease such as VHL and BHD. Technically, all RCC lesions should have been confirmed prior to performing a TAE-PCA.

## 5. Conclusions

In conclusion, we recommend the use of gelatin sponges as an embolic material over absolute ethanol when TAE is performed prior to PCA for early-stage (T1a) RCC. Gelatin sponges do not cause pain, and there was no significant difference in laboratory values between patients in whom gelatin sponge and absolute ethanol were used for TAE. At the subsequent PCA, tumor size is significantly correlated with operative time and the number of cryoneedles required. Our results will bring new knowledge around combined TAE-PCA for patients with RCC, and they also suggest the current technical limitations and medical costs of PCA for RCC lesions.

## Figures and Tables

**Table 1:** Patient characteristics.

**Sex, M/F**	18/9
**Patient age ^a^**	74 (16)
**Tumor side (right/left) ^b^**	14/14
**Tumor size (mm) ^a^**	24.8 (7.2)
**Embolization with absolute ethanol and iodized oil**	14 (F/M = 6/8)
**Embolization with gelatin sponge and iodized oil**	13 (F/M = 3/10)

^a^ Presented as the mean (standard deviation). ^b^ Includes 1 patient with 2 tumors treated in 2 stages after a single embolization. F, female; M, male.

**Table 2:** Transarterial embolization.

	Total RCC (n = 28)	Absolute Ethanol and Iodized Oil (n = 15) ^a^	Gelatin Sponge and Iodized Oil (n = 13)	*p* Value
**Tumor size (mm) ^b^**	24.8 (7.2)	23.9 (7.5)	25.7 (7.5)	NS
**Operative time (min) ^b^**	71.4 (33.2)	81.7 (39.0)	60.4 (20.3)	NS
**Number of patients who complained of pain during the procedure**	3	3	0	<0.05 (0.038)
**Iodized oil accumulation ^b,c^**	2.1 (0.7)	1.7 (0.6)	2.6 (0.6)	<0.01 (0.002)
**Vascularity (hyper/hypo)**	24/4	12/3	12/1	NS

^a^ Includes 1 patient with 2 lesions treated by 2 cycles of cryoablation following a single embolization. ^b^ Presented as the mean (standard deviation). ^c^ 1 = poor, 2 = intermediate, 3 = good.

**Table 3:** Clinical characteristics at percutaneous cryoablation and their influences on operative time.

Clinical Characteristic		*p* Value ^a^
**Operative time (min) ^b^**	172.4 (42.0)	-
**Tumor location (upper/middle/lower kidney)**	6/14/8	NS
**Hydrodissection (implemented/not implemented)**	22/6	NS
**Iodized oil accumulation by TAE (poor/fair/good)**	6/12/10	NS
**Vascularity on TAE (hyper/hypo)**	24/4	NS
**Embolic material (absolute ethanol/gelatin sponge)**	15/13	NS

^a^ Compared based on the operative time required for cryoablation in terms of each clinical characteristic (two or three groups in each). ^b^ Presented as the mean (standard deviation). NS, not significant; TAE, transarterial embolization.

**Table 4:** Correlation of tumor size with operative times and number of cryoneedles required.

Operative Time/Options		Correlation Coefficient (ρ)	*p* Value
**TAE**	**Operative time**	−0.09	NS (0.64)
**PCA**	**Operative time**	0.55	<0.01 (0.002)
	**Total number of cryoneedles**	0.78	<0.0001

NS, not significant; PCA, percutaneous cryoablation; TAE, transarterial embolization.

**Table 5:** Changes in laboratory data.

	Pre-TAE	Post-PCA	Change Between Pre-TAE and Post-PCA	Absolute Ethanol and Iodized Oil	Gelatin Sponge and Iodized Oil	*p* Value ^d^
**CRP (mg/dL)** **(n = 26) ^a,b^**	0.23 (0.56)	2.69 (2.75)	2.46 (2.65) ↑ (*p* < 0.0001)	3.37 (3.32) ↑ (n = 14)	1.40 (0.50) ↑ (n = 12)	NS
**eGFR** **(mL/min/1.73 m^2^)** **(n = 28) ^b,c^**	47.2 (22.1)	44.6 (22.8)	2.6 (8.1) ↓ (NS, *p* = 0.05)	1.8 (9.7) ↓ (n = 15)	3.5 (5.5) ↓ (n = 13)	NS
**Hb (g/dL)** **(n = 28) ^b,c^**	13.0 (1.9)	12.4 (2.1)	0.6 (0.8) ↓ (*p* < 0.001)	0.7 (0.9) ↓ (n = 15)	0.4 (0.6) ↓ (n = 13)	NS

^a^ Excluding 2 patients: no preoperative data (n = 1) and incidental femoral neck fracture after TAE (n = 1). ^b^ Presented as the mean (standard deviation). ^c^ Including 1 patient with 2 lesions treated by 2 cycles of cryoablation following a single TAE. ^d^ Comparing absolute ethanol and gelatin sponge. CRP, C-reactive protein; eGFR, estimated glomerular filtration rate; Hb, hemoglobin; PCA, percutaneous ablation; TAE, transarterial embolization.

## Data Availability

Data cannot be made available. The original IRB approval does not permit the collected data to be made public.
